# Pathological complete response achieved by gemcitabine plus cisplatin therapy for initially unresectable advanced gallbladder cancer: a case report

**DOI:** 10.1186/s40792-022-01375-z

**Published:** 2022-01-26

**Authors:** Yuya Miura, Ryo Ashida, Teiichi Sugiura, Katsuhisa Ohgi, Mihoko Yamada, Shimpei Otsuka, Akiko Todaka, Katsuhiko Uesaka

**Affiliations:** 1 Division of Hepato-Biliary-Pancreatic Surgery, Shizuoka Cancer Center, 1007 Shimonagakubo, Nagaizumi-cho, Sunto-gun, Shizuoka, 411-8777 Japan; 2 Division of Gastrointestinal Oncology, Shizuoka Cancer Center, Shizuoka, Japan

**Keywords:** Gallbladder cancer, Conversion surgery, Pathological complete response, Gemcitabine plus cisplatin therapy

## Abstract

**Background:**

Conversion surgery for initially unresectable gallbladder cancer is rarely performed due to the low response rate for systemic chemotherapy, and a pathological complete response is seldom achieved.

**Case presentation:**

A 67-year-old woman with jaundice was referred to our hospital and diagnosed with unresectable gallbladder cancer with extra-regional lymph node metastasis after examinations. After biliary decompression, gemcitabine plus cisplatin therapy was started. The tumor marker levels markedly decreased, and imaging studies revealed a reduction in the primary tumor and metastatic lymph nodes. The primary tumor and metastatic lymph node were still shrunk at 4 years after the start of gemcitabine plus cisplatin therapy, so we decided to perform conversion surgery. Gallbladder bed resection and lymph node dissection were performed. The pathological findings of the resected specimen showed only partial fibrosis in the gallbladder wall and no malignant findings in the dissected lymph nodes, indicating a pathological complete response. As of 24 months after the operation, she is alive without recurrence.

**Conclusion:**

Although there have been only a few reports of conversion surgery for initially unresectable gallbladder cancer, it may be worthwhile to perform chemotherapy with the potential goal of subsequent conversion surgery.

## Background

Surgical resection is a treatment for gallbladder cancer (GBC) most likely to achieve a cure, but early detection of GBC is difficult due to its asymptomatic nature in the early stage [1]. Although the current standard treatment is chemotherapy comprising gemcitabine plus cisplatin (GC) for patients with unresectable biliary malignancy, the response rate is low, with a complete response (CR) seldom achieved [2, 3]. Conversion surgery was therefore rarely performed after effective chemotherapy, whereas it has been performed recently for cases responding to chemotherapy [4].

We herein report a case in which GC therapy was significantly effective for initially unresectable GBC, and conversion surgery was able to be performed, with a pathological CR eventually achieved.

## Case presentation

**Fig. 1:**
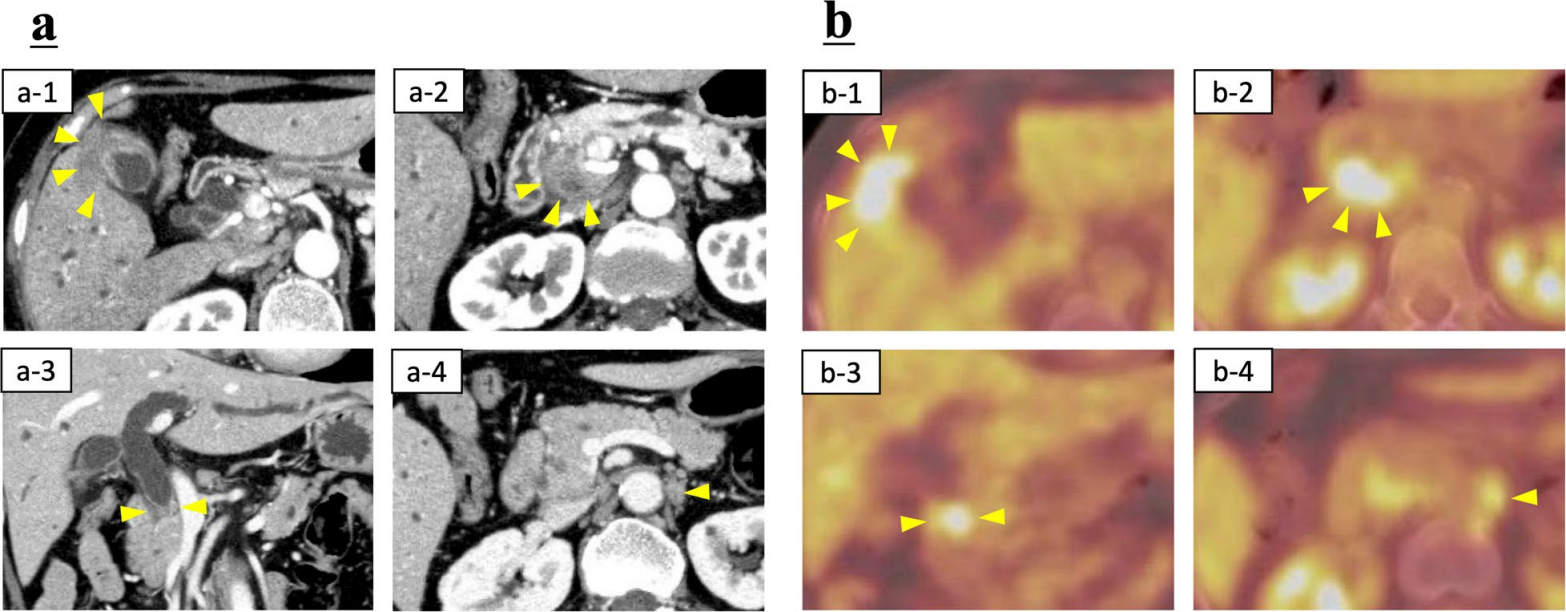
**a** Multidetector-row computed tomography findings, and the lower row shows the corresponding positron emission tomography. **a-1** The tumor at the fundus of the gallbladder infiltrating the liver parenchyma. **a-2** Bulky metastasis of the hepatoduodenal mesenteric lymph node. **a-3** Bile duct stenosis due to bile duct infiltration of the metastasis lymph node, along with dilation of the intrahepatic and extrahepatic bile ducts. **a-4** Metastasis of the para-aortic lymph node. **b** Positron emission tomography findings corresponding to **a**

**Fig. 2:**
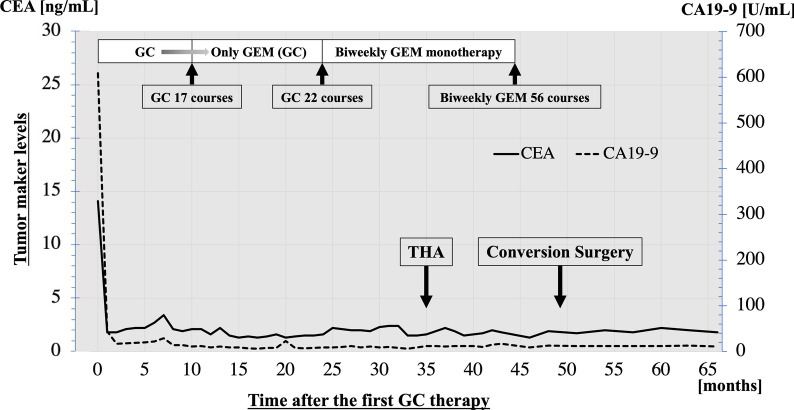
A time series showing the course of treatment and the changes in tumor markers from the start of chemotherapy. *GC* gemcitabine plus cisplatin, *GEM* gemcitabine, *CEA* carcinoembryonic antigen, *CA19-9* carbohydrate antigen 19-9, *THA* total hip arthroplasty

**Fig. 3:**
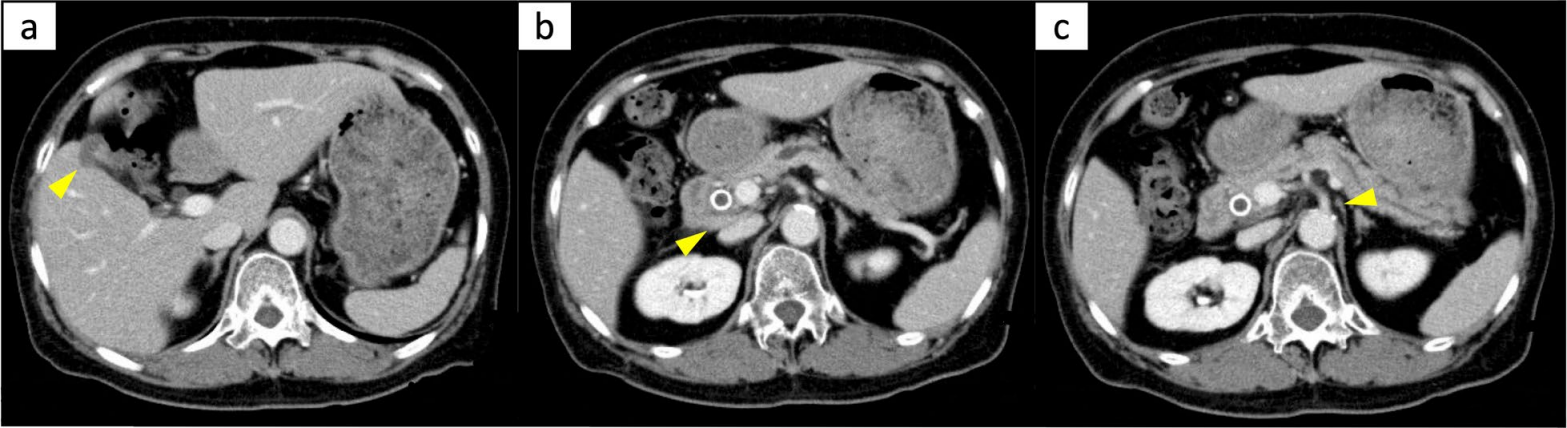
Multidetector-row computed tomography findings after two courses of gemcitabine plus cisplatin therapy. **a** The shrunken tumor at the fundus of the gallbladder. **b** The shrunken metastasis lymph node in the hepatoduodenal mesentery. **c** The shrunken para-aortic lymph node

**Fig. 4:**
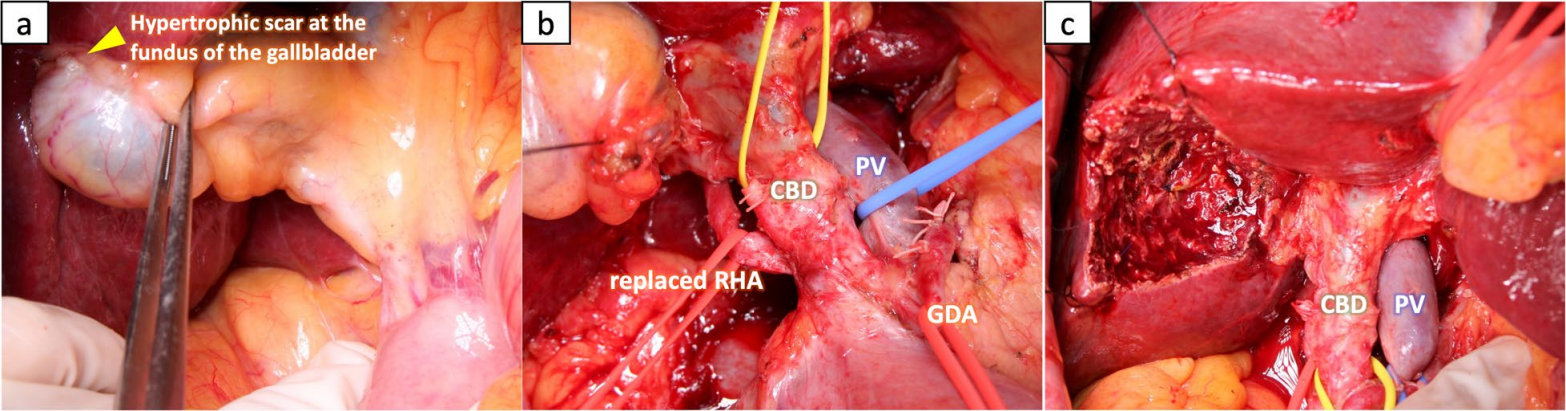
Operative findings. Gallbladder bed resection and the hepatoduodenal mesenteric lymph node dissection (extrahepatic bile duct preservation). **a** Hypertrophic scar at the fundus of the gallbladder. **b** Hepatoduodenal mesenteric lymph node dissection was performed. **c** Gallbladder bed resection was performed. *CBD* common bile duct, *RHA* right hepatic artery, *GDA* gastroduodenal artery, *SMA* superior mesenteric artery, *CHA* common hepatic artery, *GDA* gastroduodenal artery, *PV* portal vein

A 67-year-old woman with obstructive jaundice was referred to our hospital. Laboratory data on admission showed elevated levels of serum total bilirubin at 15.5 mg/dL, γ-glutamyltranspeptidase at 1347 IU/L, carcinoembryonic antigen at 14.1 ng/mL, and carbohydrate antigen 19-9 at 609 U/mL. Multidetector-row computed tomography (MDCT) revealed the tumor at the fundus of the gallbladder infiltrating the liver parenchyma (Fig. 1a-1) and bulky lymph node metastases on the posterior surface of the pancreatic head infiltrating the common bile duct (Fig. 1a-2 and a-3). The para-aortic lymph nodes were also swollen (Fig. 1a-4). Positron emission tomography showed the accumulation of fluorodeoxyglucose in the tumor and lymph nodes, including the para-aortic lymph nodes (Fig. 1b). Therefore, although pathological confirmation was not obtained, the diagnosis of unresectable GBC was made based on the markedly elevated tumor markers and obvious imaging findings. A percutaneous transhepatic bile duct stent was placed for biliary decompression, and the carcinoembryonic antigen level was 10.9 ng/mL, and the carbohydrate antigen 19-9 level was 599 U/mL after improvement of jaundice.

Systemic chemotherapy with the GC regimen (gemcitabine: 1000 mg/m^2^, days 1 and 8; cisplatin: 25 mg/m^2^, days 1 and 8; 1 course for 21 days) was started, and the subsequent treatment process is summarized in Fig. 2. One month after the start of GC therapy, the tumor marker levels had markedly decreased, and MDCT revealed a reduction of the primary tumor and metastatic lymph nodes (Fig. 3). The dose of cisplatin was gradually reduced due to adverse events of renal dysfunction and peripheral neuropathy, and gemcitabine alone was administered from 10 months after the start of GC therapy. Chemotherapy was then switched to biweekly gemcitabine monotherapy due to repeated anemia. In addition, steroid-induced osteonecrosis of the femoral head occurred, and right total hip arthroplasty was performed 35 months after the start of GC.

**Fig. 5:**
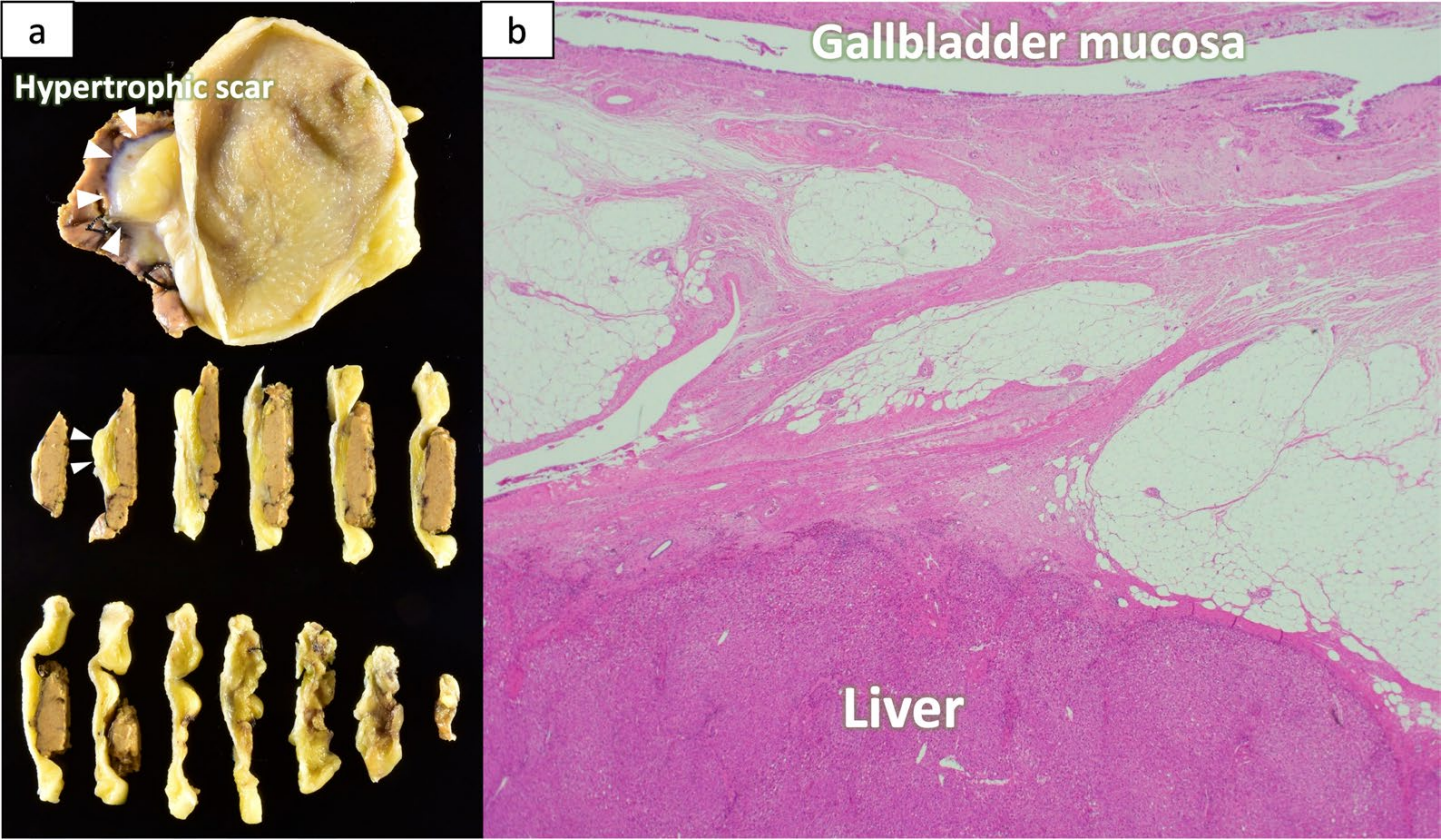
**a** Resected specimen, macroscopic findings. No lesions of the gallbladder mucosa. **b**, **c** Resected specimen, microscopic findings. No obvious malignant findings (stain, hematoxylin and eosin; magnification × 40)

Computed tomography, abdominal ultrasonography and positron emission tomography performed 4 years after the start of GC therapy showed that the primary tumor and metastatic lymph nodes remained shrunken without the accumulation of fluorodeoxyglucose, so we decided to perform conversion surgery. At 49 months after the start of GC therapy, gallbladder bed resection and regional lymph node dissection (with preservation of the extrahepatic bile duct) and sampling of the para-aortic lymph nodes were performed (Fig. 4). All of the para-aortic lymph nodes sampled for intraoperative frozen section examinations were negative for cancer. The posterior pancreatic lymph nodes that initially infiltrated the common bile duct (Figs. 1a-2 and 3b) were able to be dissected from the bile duct. The pathological findings of the resected specimen, including the entire part of the gallbladder, showed only partial fibrosis in the gallbladder wall and no malignant findings in the dissected lymph nodes, resulting in a pathological CR (Fig. 5). Adjuvant chemotherapy was not performed. As of 24 months after the operation, she is alive without recurrence.

## Discussion

Conversion surgery for initially unresectable biliary tract cancer is rarely performed due to the low response rate for systemic chemotherapy [2, 3], and a pathological complete response is seldom achieved. CR is even more rarely achieved among patients with advanced GBC who have completed chemotherapy [5–8], and curative resection after effective chemotherapy for initially unresectable GBC was rarely performed. The present case is therefore very valuable, as GC therapy was markedly effective, allowing subsequent conversion surgery to be performed, which resulted in a pathological CR.

Some previous reports have described cases that were initially diagnosed with unresectable GBC, but were able to undergo radical resection after chemotherapy, as shown in Table 1 [7–15]. According to those previous reports, only 3 of 12 cases achieved a pathological CR. The median interval from the first chemotherapy session to conversion surgery was 7.5 months (range 6–36 months), whereas the interval in the present case was 49 months. As there was no consensus between medical oncologists and surgeons concerning conversion surgery for unresectable GBC at the time in our institution, the interval to conversion surgery was relatively long. There have been no studies concerning the optimal duration of chemotherapy for conversion surgery in initially unresectable biliary tract cancer. Although a different disease, a previous report showed that the overall survival of patients with initially unresectable pancreatic cancer who underwent conversion surgery 8 months or longer after the first chemotherapy session was significantly better than that of those treated for less than 8 months [16], which may be a useful indicator. Taking these results into account [7–16], conversion surgery may be able to be considered in cases responding to chemotherapy with a partial response or CR on computed tomography and negative findings on positron emission tomography as well as decreased tumor marker levels after at least 7–8 months from the first chemotherapy session. Medical oncologists and surgeons should communicate closely during this period, considering the possibility of conversion surgery.

**Table 1:** The cases diagnosed initially with an unresectable gallbladder cancer and could be performed radical resection

No	Author reference (year)	Age (yeaears)	Reasons for unresectability	Chemotherapy	*Interval (months)	Surgery	Residual tumor status	Histological evaluation	Postoperative survival (months)	Status
1	Morimoto et al. [9] (2008)	69	Liver metastasis	GEM	12	GBR, partial resection of the duodenum and the colon, partial liver resection of segment 8, lymphadenectomy	R0	PR	20	Alive
2	Sharma et al. [5] (2010)	N/A	N/A	GEM + oxaliplatin	N/A	N/A	N/A	N/A	N/A	N/A
3	Takita et. al [10] (2011)	57	Metastasis of para-aortic lymph node	GEM + S-1	9	GBR, lymphadenectomy	R0	PR	12	Alive
4	Kato et al. [11] (2013)	57	Arterial infiltration portal vein infiltration	GEM	N/A	Right hemi-hepatectomy with caudal lobectomy, EBDR, lymphadenectomy	R0	N/A	42	Alive
5	Kato et al. [11] (2013)	57	Arterial infiltration	GEM	N/A	Right hemi-hepatectomy with caudal lobectomy, EBDR, lymphadenectomy	R1	N/A	18	Dead
6	Kato et al. [11] (2013)	57	Arterial infiltration	GEM	N/A	Hepatectomy of segment 4a and 5, EBDR, lymphadenectomy	R1	N/A	19	Dead
7	Kato et al. [11] (2013)	61	Arterial infiltration, duodenal infiltration	GEM	N/A	Hepatectomy of segment 4a and 5, EBDR, lymphadenectomy	R1	N/A	8	Dead
8	Einama et al. [12] (2014)	60	Arterial infiltration	S-1	36	Right hemi-hepatectomy, EBDR, lymphadenectomy	R0	PR	30	Alive
9	Rama et al. [13] (2014)	64	Metastasis of para-aortic lymph node	GEM + CDDP	6	GBR, EBDR, lymphadenectomy	R0	PR	6	Alive
10	Kato et al. [14] (2015)	62	Arterial infiltration	GEM + CDDP	9	Right hepatic trisegmentectomy, with caudal lobectomy, EBDR, Lymphadenectomy	R0	PR	N/A	Dead
11	Tsuyuki et al. [15] (2018)	76	Metastasis of para-aortic lymph node	GEM + CDDP	6	GBR, lymphadenectomy	R0	PR	7	Alive
12	Hashimoto et al. [7] (2019)	47	Local advanced	GEM + CDDP	6	GBR, lymphadenectomy	R0	CR	14	Alive
13	Kato et al. [8] (2020)	59	Metastasis of para-aortic lymph node	GEM + CDDP	6	GBR, EBDR, lymphadenectomy	R0	CR	11	Alive
14	Present case	71	Metastasis of para-aortic lymph node	GEM + CDDP	49	GBR, lymphadenectomy	R0	CR	18	Alive

*N/A* not available, *GEM* gemcitabine, *PR* partial response, *S − 1*, tegafur/gimeracil/oteracil, *GBR* gallbladder bed resection, *EBDR* extrahepatic bile duct resection, *SD* stable disease, *CDDP* cisplatin, *CR* complete response

*Interval to surgery from the first chemotherapy

To our knowledge, the achievement of a pathological CR for advanced biliary tract cancer has been reported with only eight cases (Table 2). According to those previous reports [5–8, 17–20], four of the eight cases had GBC. The median interval from the first chemotherapy session to conversion surgery was relatively short, at 6 (range 2–24) months, implying a relatively rapid response to chemotherapy rather than a gradual anti-tumor effect following prolonged use of chemotherapy. Only two cases received adjuvant chemotherapy and recurrence was observed in just one case. That only one out of nine cases showed recurrence may be evidence of completely systemic tumor control with chemotherapy. In the previous phase II trial of systemic chemotherapy with gemcitabine plus oxaliplatin regimen for unresectable GBC [5], only 1 case out of 48 was able to undergo conversion surgery, and that case achieved a pathological CR. Even in this small study, this result implies that responders to chemotherapy, even when initially assessed as unresectable, should be evaluated for conversion surgery. Responders to chemotherapy with pathological CR may have a different genetic profile and therefore responded well. Accumulating more cases involving conversion surgery performed for initially unresectable biliary tract cancer will be necessary to determine the likelihood of performing such surgery in patients responding remarkably to chemotherapy, given the potential publication biases in previous reports.

**Table 2:** The cases achieved a pathological complete response for advanced biliary tract cancer

No	Author reference (year)	Age (years)	Sex	Disease			Preoperative chemotherapy		*Interval (months)		Postoperative Survival (months)		Adjuvant chemotherapy		Recurrence, Status	
1	Sharma et al. [5] (2010)	N/A	N/A	GBC			GEM + oxaliplatin		N/A		N/A		N/A		N/A, N/A	
2	Moussata et al. [6] (2012)	58	F	GBC			GEM + oxaliplatin		5		14		None		Local recurrence (lymph node metastasis), Alive	
3	Lim et al. [17] (2013)	58	M	Extrahepatic cholangiocarcinoma			GEM + S − 1		7		3		None		None, alive	
4	Walker et al. [18] (2014)	64	M	Extrahepatic cholangiocarcinoma			GC		2		18		None		None, alive	
5	Hashimoto et al. [7] (2014)	47	F	GBC			GC		6		14		S-1		None, alive	
6	Kato et al. [8] (2014)	59	F	GBC			GC		6		11		None		None, alive	
7	Matsubara et al. [19] (2016)	68	F	Hilar cholangiocarcinoma			GC + S-1		6		9		None		None, alive	
8	Watanabe et al. [20] (2017)	70	F	Extrahepatic cholangiocarcinoma			GEM + S − 1		24		48		5 courses of GC		None, alive	
9	Present case	71			F	GBC		GC		49		24		None		None, alive

*GBC* gallbladder cancer, *GEM* gemcitabine, *GC* gemcitabine plus cisplatin, *S − 1* tegafur/gimeracil/oteracil

*Interval to surgery from the first chemotherapy

Advanced GBC often require highly invasive surgery, such as major hepatectomy, pancreatoduodenectomy, extrahepatic bile duct resection, or hepatopancreatoduodenectomy, in accordance with the tumor location and extension [21, 22]. However, when choosing the surgical procedure, R0 resection with sufficient safety is required. In the present case, if we had attempted resection of the primary tumor and metastatic lymph node metastases prior to chemotherapy, gallbladder bed resection and pancreatoduodenectomy with para-aortic lymph node dissection would have been needed. However, gallbladder bed resection and regional lymph node dissection (with extrahepatic bile duct preservation) along with sampling of the para-aortic lymph nodes were performed as conversion surgery, as the present patient with renal disfunction and other unfavorable conditions was a high-risk patient, and R0 resection was considered feasible based on the intraoperative findings. Although there is no clear rationale concerning whether or not the entire area where the tumor was initially present should have been resected, the extrahepatic bile ducts were able to be preserved based on intraoperative findings in the present case, and a 2-year recurrence-free survival was achieved. This result suggests that conversion surgery that preserves the area from which the tumor has disappeared may be acceptable.

## Conclusion

Although there have been only a few reports of conversion surgery for initially unresectable GBC, it may be worthwhile to perform chemotherapy with the potential aim of performing subsequent conversion surgery. Accumulating more cases will be necessary to determine the optimal chemotherapy, timing of surgery, and surgical method.

## Data Availability

The datasets used during the current study are available from the corresponding author on reasonable request.

## Abbreviations

GBC: Gallbladder cancer
GC: Gemcitabine plus cisplatin
CR: Complete response
MDCT: Multidetector-row computed tomography
